# Editorial

**DOI:** 10.1002/1878-0261.12613

**Published:** 2020-01-06

**Authors:** Julio E. Celis

## Abstract

May 2020 be a rewarding year of change. The recent commitment of the European Commission and policymakers to fight cancer in partnership is expected to bring forward the changes long required in the field. *Molecular Oncology* will stay at the forefront of all developments in the area of oncology by providing a flexible platform for European and International cancer research.

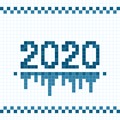

Last year, Molecular Oncology achieved several landmarks. Increased recognition of the standing of the journal has driven the submission of around 1200 manuscripts in 2019, and, with the valuable contribution of the journal’s Section Editors, we have published over 150 papers, carefully selected for their novelty and quality. Among these, we are proud to showcase two thematic issues: one on https://febs.onlinelibrary.wiley.com/toc/18780261/2019/13/1 https://febs.onlinelibrary.wiley.com/toc/18780261/2019/13/1 and one on the essential components of a pan‐European, mission‐oriented, cancer policy that would be required to https://febs.onlinelibrary.wiley.com/toc/18780261/2019/13/3. Nevertheless, we have also experienced a loss, as José Manuel Moreira, who has worked as Editorial Manager of *Molecular Oncology* for 12 years and was lately acting as Reviews Editor, decided to leave the journal to concentrate on his duties as Full Professor at the University of Copenhagen. We very much appreciate the work José has done for the journal, since we would not be where we are today without his efforts. We wish him all the best in his future activities.

Keeping up with the development of the journal, the FEBS Publication Committee appointed Professor Kevin Ryan from the Cancer Research UK Beatson Institute in Glasgow as Joint Editor‐in‐Chief of *Molecular Oncology,* starting on January 1^st^, 2020. I am delighted to welcome Kevin to the journal team. Kevin is an internationally renowned cancer biologist working in the areas of cell death, metabolism and autophagy, with a strong focus on both basic and translational cancer research, which are critical priorities for the journal. Also, he has extensive publishing experience gathered over the years in senior editorial positions (see short biography in Box 1).

Box 1 **Biography of Professor Kevin Ryan**

Professor Kevin Ryan is a cancer biologist and world leader in the fields of cell death, metabolism and autophagy. Following his PhD, Professor Ryan conducted postdoctoral studies at the US National Cancer Institute. He then obtained a Cancer Research UK Senior Fellowship to establish his laboratory at the Beatson Institute for Cancer Research in Glasgow. His career has grown considerably ever since, as marked by 115 publications. Importantly, his findings are currently being translated towards cancer early detection approaches and a clinical trial. He is frequently invited to speak at international research institutes and at prestigious meetings, including Keystone symposia, European Cancer Organisation (ECCO) meetings and Gordon conferences. He has also been involved in the organization of several high‐profile scientific conferences, acting as Chair of the 2019 EMBO Conference on Autophagy, The Beatson International Cancer Conference and ‘Genes and Cancer’ – the UK’s longest‐running cancer conference. As a result of these collective achievements and activities, Professor Ryan has received several awards, most notably the ‘EACR Cancer Research Award’ – a highly revered award of the European Association for Cancer Research – and the 2012 Tenovus Medal, which is presented annually to a scientist with a Scottish link. Professor Ryan is also an elected Fellow of the Royal Society of Edinburgh.

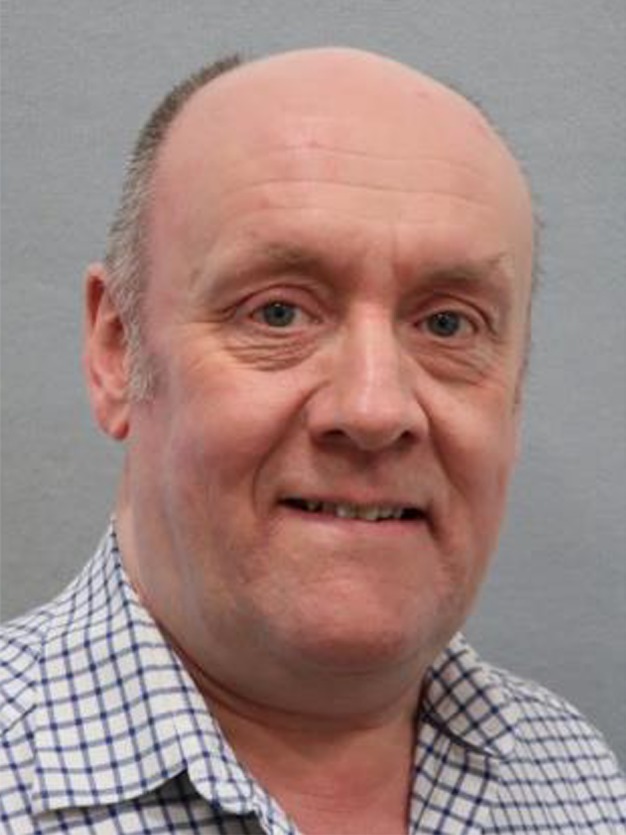

Indicative of Professor Ryan’s standing in the field are his memberships in scientific advisory boards and journal editorial boards. Notably, he has served as Associate Editor on *Autophagy*, Commissioning Editor on *the FEBS Journal* and Deputy Editor on *Oncogene*. Professor Ryan is excited to be joining *Molecular Oncology*, which – being an Open Access journal of FEBS Press – serves the scientific community through both the broad dissemination of cutting‐edge research and the investment of its full income on the funding young researchers.

As Joint Editors‐in‐Chief, Kevin and I will work closely with the Section Editors, Senior Editors and the new Editorial Manager to oversee the direction, scope and editorial policy that will guarantee the sustained quality of the journal. Moreover, given the increased number of submissions, one of our main priorities will be to ensure the fast handling of incoming manuscripts. Also, we plan to introduce several new article types in order to provide an extended platform for basic/preclinical, early clinical and prevention research, computational analyses of clinical/prevention and biological data, science policy initiatives and more. To start with, we have just introduced Commentaries to initiate discussions on the implications of selected research articles published in the journal (see Commentary in this issue). Commentaries are short (max. 1000 words) articles that highlight advances in knowledge and specific features of selected papers published in the journal.

I am looking forward to this year, with the assurance that it will be a rewarding year of change. Importantly, the recent commitment of the European Commission and policymakers to fight cancer in partnership by sharing expertise, resources and data is expected to bring forward the changes long required in the field. *Molecular Oncology* will stay at the forefront of all developments in the area of oncology by providing a flexible platform for European and International cancer research.

